# Transforming long-term adjunctive therapy for cognitive impairment: the role of multimodal self-adaptive digital medicine

**DOI:** 10.3389/fneur.2025.1571817

**Published:** 2025-04-01

**Authors:** Dong Wen, Yihao Xing, Yiduo Yao, Guangjin Liang, Yijie Xing, Tzyy-Ping Jung, Hao Yu, Xueguang Xie, Xianglong Wan, Tiange Liu, Dingna Duan, Danyang Li, Yanhong Zhou

**Affiliations:** ^1^School of Intelligence Science and Technology, University of Science and Technology Beijing, Beijing, China; ^2^The Key Laboratory for Brain Computer Intelligence and Digital Therapy of Hebei Province, University of Science and Technology Beijing, Beijing, China; ^3^Department of Electrical and Electronic Engineering, Faculty of Engineering, University Putra Malaysia, Serdang, Selangor, Malaysia; ^4^School of Energy Resources, China University of Geosciences (Beijing), Beijing, China; ^5^Swartz Center for Computational Neuroscience, University of California, San Diego, San Diego, CA, United States; ^6^School of Information Science and Engineering, Yanshan University, Qinhuangdao, China

**Keywords:** digital medicine, self-adaptive, multimodal, cognitive impairment, long-term adjunctive therapy

## 1 Introduction

Cognitive impairment [CI; (1)], particularly dementia, poses a growing challenge to global public health as the aging population continues to expand. Traditional treatments for CI often fail to provide lasting improvements, leaving a critical gap in effective long-term management (2). Despite ongoing developments in new therapies, we believe that current approaches, including pharmacological interventions, lack the sustainability needed to impact long-term cognitive function meaningfully. Therefore, the need for more effective long-term adjunctive therapy (LAT)—which refers to the long-term use of non-drug therapies alongside primary treatments (typically medication) to manage symptoms and enhance the quality of life (QoL)—is more urgent than ever (3, 4).

In recent years, we have become increasingly convinced that integrating multimodal interventions (MMI), self-adaptive systems (SS), and digital medicine (DM) holds the potential to transform the landscape of CI treatment fundamentally. While MMI, SS, and DM are promising individually, their true power lies in their synergy. MMI (5) involves employing two or more complementary strategies to tackle multiple modifiable risk factors known to contribute to cognitive decline. SS (6) is a closed-loop system with a feedback mechanism to optimize intervention strategies based on patient feedback and environmental changes. DM (7) refers to applying digital technologies and tools to enhance medical practice and deliver more precise and personalized healthcare services.

In this opinion piece, we propose that the integration of multimodal self-adaptive digital medicine (MSDM) enhances the effectiveness of current treatments and provides a pathway for developing more personalized, sustainable solutions. By highlighting the combined potential of these technologies, we hope to spark further discussion on their future application in the LAT of CI.

## 2 The status of adjunctive therapy for CI

### 2.1 The potential and limitations of MMI in LAT

In our opinion, the multifaceted nature of CI requires a treatment approach that is equally comprehensive and dynamic. While traditional interventions often fail to address the complexity of CI, MMI has emerged as a promising solution, particularly for LAT (8, 9). MMI integrates diverse therapeutic strategies, ranging from cognitive training to physical therapy (10–14) to address various facets of CI (5). The real strength of MMI lies not in any single intervention but in its ability to tailor a combination of strategies to each patient's unique needs.

However, challenges remain in ensuring that MMI can be consistently implemented to match the evolving needs of patients. While MMI has demonstrated significant improvements in cognitive function, daily activities, and overall QoL (15), its success is contingent on the system's ability to adapt to the dynamic needs of the patient over time. This adaptability is one of MMI's key benefits and its most significant challenges. For instance, while some interventions might offer immediate benefits, maintaining engagement over the long term is often tricky. The structured nature of MMI can lead to treatment fatigue, particularly if patients are required to engage in multiple diverse therapeutic activities (16–18). This is why we believe the future of MMI depends on its ability to be more responsive to individual patient needs, potentially with the help of SS, which can adjust therapy based on patient feedback.

Despite these challenges, the potential of MMI in LAT cannot be ignored. Recent trends have shown that multimodal cognitive and behavioral interventions have comparable or even more significant effects on cognition than pharmacological treatments (19). To truly unlock its benefits, future research must focus on improving its personalized, adaptive nature and ensuring that interventions remain engaging and sustainable over time (5).

### 2.2 The role of SS in adapting therapies for CI

SS represents one of the most exciting developments in the LAT of CI. Traditional treatments for CI often struggle to meet patients' dynamic and multifactorial needs, but SS promises to adapt therapy to real-time individual responses (20, 21). This adaptability—particularly in long-term care settings—allows for continuous treatment adjustment based on disease progression, emotional fluctuations, and adherence (6). In our opinion, this is one of SS's most important advantages, as it offers flexibility often lacking in more rigid, one-size-fits-all treatment approaches.

Despite this potential, the widespread implementation of SS in LAT faces significant challenges. One key issue is the need for real-time data collection and analysis (22, 23), which demands considerable technological infrastructure. In resource-limited environments, the lack of access to necessary technologies and expertise could hinder the implementation of SS, limiting its potential. Moreover, while SS can offer dynamic adaptability, this may also introduce complexity for patients and caregivers, potentially reducing compliance.

That said, we believe the future of SS depends on simplifying these systems and ensuring they are easy to use in everyday settings. Moving forward, advancements in artificial intelligence (AI) and machine learning will help overcome these barriers, enabling SS to become an integral part of LAT for CI.

### 2.3 Revolutionizing CI treatment through DM

DM has rapidly gained traction in the LAT of CI. In our opinion, DM constitutes a revolutionary shift in how we approach CI treatment. Unlike traditional methods, which often offer short-term relief, DM leverages cutting-edge technologies such as mobile apps, wearables, and brain-computer interfaces (BCI) to provide continuous, real-time monitoring and personalized therapy (7, 24–27). By harnessing AI, cloud computing, and big data, DM facilitates immediate symptom alleviation and provides a means to adjust interventions based on the patient's evolving needs dynamically.

One of the key advantages of DM is its ability to provide continuous feedback and adapt interventions in real time, something traditional therapies cannot achieve. In our opinion, this flexibility makes DM an essential component of LAT (28, 29). Recent trends have shown that DM technology is a promising approach to supporting healthcare providers and family caregivers caring for patients with cognitive impairment (30, 31). Through tools such as cognitive training (CT) apps and lifestyle modification platforms, DM not only stimulates neural circuits but also fosters neuroplasticity—offering patients a way to combat cognitive decline over time (32, 33). This ongoing stimulation and adaptation can significantly mitigate the long-term healthcare burden typically associated with CI, potentially improving cognitive function and overall QoL for patients (34).

Despite these advancements, significant challenges remain. For one, integrating DM into the everyday lives of patients necessitates overcoming barriers to accessibility, particularly for older adults or those in resource-limited settings. Additionally, while DM has shown promise in short-term symptom alleviation, its full potential in long-term cognitive enhancement and maintenance remains underexplored. We believe that future research must focus on optimizing DM tools to ensure their long-term effectiveness and to refine their integration with other therapeutic modalities.

## 3 Our opinion

### 3.1 The integration of MMI, SS, and DM transforms LAT for CI

In our opinion, the integration of MSDM presents an unparalleled opportunity to overcome the limitations of current CI therapies. While the individual benefits of MMI, SS, and DM are widely recognized, we believe that their synergistic potential could fundamentally change the landscape of LAT. A study (35) employed a brain plasticity-based self-adaptive CT game developed through leisure-time physical activity (LTPA) to conduct multimodal cognitive function training (MCFT) for individuals with amnestic mild cognitive impairment (aMCI) and mild dementia. This study found it effective in improving cognitive function, working memory, attention, and coordination, with no side effects reported. The positive effects of this multimodal cognitive function training were sustained for 1 year after the intervention. Recent studies (36–40) have also indicated that the integration of MMI and DM offers long-term benefits in cognitive function, activity levels, and QoL for older adults at risk of cognitive decline. By combining these three approaches, we can develop more personalized, dynamic, and sustainable treatments for CI that will significantly improve patient outcomes (41).

We are particularly excited about the possibilities that emerge when these technologies are integrated. For example, MMI provides a wide range of therapeutic strategies. However, its full potential can only be realized when paired with SS, which can dynamically adjust interventions based on patient needs. Furthermore, DM can provide real-time monitoring, allowing for continuous, data-driven adjustments to the treatment plan. Together, these technologies create a feedback loop that ensures therapy remains relevant and effective as the patient's condition evolves.

While research in this area is still in its early stages, we believe the future is bright. Early studies have shown promising results, such as improved cognitive function, memory, and QoL in individuals with aMCI and mild dementia (35). However, much work remains to be done. We believe future research should focus on refining these systems, improving their integration, and ensuring they are both scalable and accessible. When optimized, the combination of these three approaches, may offer long-term benefits that transform the care of CI patients.

### 3.2 A promising framework in CI adjunctive therapy

**Figure 1:**
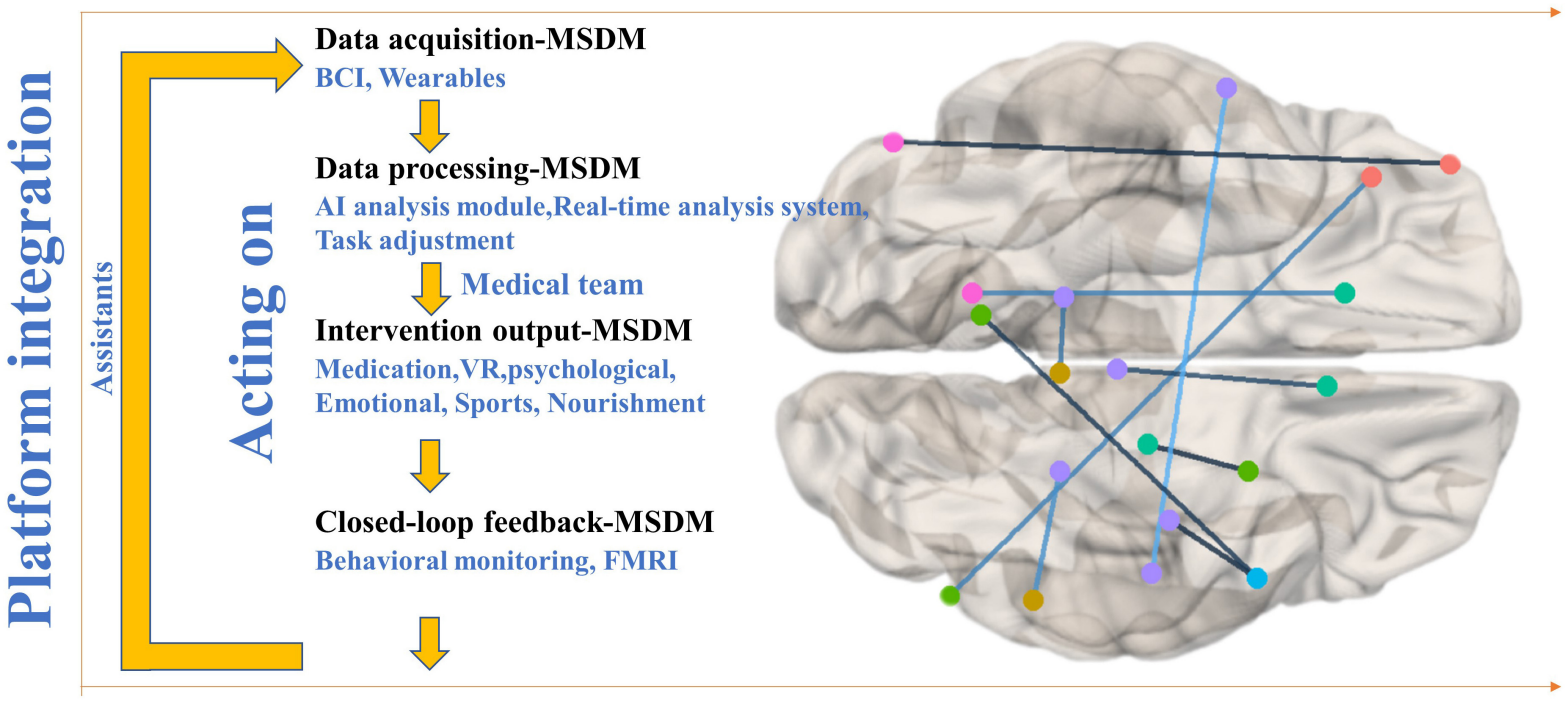
Schematic representation of the MSDM model architecture for closed-loop adaptive therapy in cognitive impairment.

This paper proposes a promising framework integrating MMI, SS, and DM for LAT in CI. The MSDM model consists of four layers—data acquisition, processing, intervention output, and closed-loop feedback—forming a continuously optimized treatment cycle (Figure 1).

The data acquisition layer captures real-time brain activity, behavioral patterns, and physiological parameters, providing a comprehensive assessment of cognitive and emotional states. In the model schematic (Figure 1), the lines connecting different brain regions represent functional connectivity or information transfer between distinct cerebral areas. These neurophysiological connections are critically associated with cognitive functions, emotional processing, and motor control regulation, particularly in LAT interventions for patients with CI disorders. EEG biomarkers offer insights into neural function, while behavioral and physiological indicators complement these insights, forming the sensory foundation for subsequent processing. The data processing layer employs AI-powered SS to analyze multimodal data dynamically. For example, if EEG signals indicate reduced attentional engagement, the system enhances cognitive stimulation through task prompts or music therapy. Psychological and emotional therapies (e.g., relaxation techniques, music therapy) are adjusted based on self-reported mood data and physiological stress markers, ensuring personalized, real-time adaptation to optimize engagement and therapeutic outcomes.

The intervention output layer integrates DM technologies to deliver personalized therapies, including virtual reality (VR)-based CT for memory and executive function, brain stimulation [e.g., transcranial direct current stimulation (tDCS), repetitive transcranial magnetic stimulation (rTMS)] guided by EEG and BCI feedback, and behavioral interventions using wearables and mobile apps to monitor activity and lifestyle. The closed-loop feedback layer ensures continuous optimization by tracking neural, behavioral, and physiological responses, dynamically adjusting interventions, and creating an adaptive therapeutic cycle. Longitudinal monitoring of EEG stability (e.g., sustained α-band activity), behavioral response times, and emotional fluctuations provide quantitative measures of cognitive and psychological improvement.

Additionally, the feedback system is seamlessly integrated into digital health platforms, facilitating remote collaboration among caregivers and healthcare professionals. AI-driven virtual assistants enhance treatment adherence, and a collaborative digital platform connects neurologists, psychologists, and therapists, fostering interdisciplinary coordination to refine treatment strategies and improve patient outcomes.

## 4 Challenges in the LAT of CI with MSDM

While the integration of MSDM holds great promise for enhancing LAT in CI, several challenges must be addressed before this model can reach its full potential. In our opinion, overcoming these obstacles will require careful attention to both technological and human factors.

### 4.1 Limited long-term longitudinal evaluation and large-scale empirical validation

Currently, there are few studies on long-term assisted care for cognitive impairment, and large-scale, cross-center longitudinal studies, combined with Real-World Evidence (RWE), are needed to determine the actual value of digital interventions in chronic, progressive cognitive impairment. Digital health often relies on various hardware and software platforms, but there is no unified standard (42). For elderly or cognitively impaired patients, complex hardware operations can reduce compliance (43). Cross-platform data standardization and device interoperability protocols must be promoted to achieve an integrated “seamless” user experience while simplifying the user interface and lowering the technical barriers for patients and caregivers.

### 4.2 Insufficient deep integration of data and intervention models

There is a wide variety of data in the long-term management of CI. However, there is a lack of in-depth understanding of the interrelationships among these different data streams. For example, the combining music therapy and VR scenes may produce superimposed effects at the EEG level. However, most current approaches are based on single-modal analysis, ignoring MMI effects (5). For multiple interventions to be linked, it is necessary to identify the optimal timing of each intervention and its dependence on timing.

### 4.3 Poor adaptability of SS to specific subgroups

There is still a need to develop adaptive systems for different disease subtypes and stages that can automatically identify the course of a patient's disease and implement layered interventions to avoid “one size fits all” (44). For example, machine learning can dynamically switch the type or intensity of intervention mode after identifying disease course characteristics. Most adaptive systems focus on cognitive parameters and ignore the patient's emotional needs, social support, and cultural background. In music therapy, the choice of song and rhythm can be counterproductive if it conflicts with the patient's past cultural memories or emotional connections (45, 46).

## 5 Conclusion

In summary, this study explores the current state of LAT for CI and highlights the significant potential of integrating MMI, SS, and DM in addressing the complex needs of patients with CIs. Additionally, we identify the challenges and future directions of the MSDM framework. While concerns remain regarding data privacy, data analysis, and the diversity of patient needs, the combination of MMI's multifunctional advantages, SS's flexibility, and DM's enhanced accessibility demonstrates potential to overcome current limitations. This integrated approach offers a promising pathway for future research and clinical practice in LAT for CI.
